# Effects of Radiofrequency Diathermy Plus Therapeutic Exercises on Pain and Functionality of Patients with Patellofemoral Pain Syndrome: A Randomized Controlled Trial

**DOI:** 10.3390/jcm12062348

**Published:** 2023-03-17

**Authors:** Manuel Albornoz-Cabello, Alfonso Javier Ibáñez-Vera, Cristo Jesús Barrios-Quinta, Inmaculada Carmen Lara-Palomo, María de los Ángeles Cardero-Durán, Luis Espejo-Antúnez

**Affiliations:** 1 Department of Physiotherapy, University of Seville, 41004 Seville, Spain; 2 Department of Health Sciences, University of Jaen, 23071 Jaén, Spain; 3 Department of Physiotherapy, Andalusian Health Service, 41071 Seville, Spain; 4 Department of Nursing, Physical Therapy and Medicine, University of Almeria, 04120 Almeria, Spain; 5 Department of Medical-Surgical Therapy, Faculty of Medicine and Health Sciences, University of Extremadura, 06006 Badajoz, Spain

**Keywords:** diathermy, functionality, patellofemoral pain syndrome, pain, radiofrequency

## Abstract

Although consensus has been reached about the use of therapeutic exercise in patellofemoral pain syndrome, several techniques used worldwide such as radiofrequency diathermy could be useful as complementary therapy. The objective of this randomized controlled trial was to compare the effects of adding radiofrequency diathermy to therapeutic exercises in patients with patellofemoral pain syndrome. Fifty-six participants were randomly assigned either to radiofrequency diathermy plus therapeutic exercises group (n = 29) or therapeutic exercises group (n = 27). Both groups received the same therapeutic exercises, and the diathermy group additionally received monopolar dielectric diathermy for three weeks (5–3–2 weekly sessions). Data related to intensity of pain, probability of neuropathic pain, functionality, and range of movement of the knee were measured at baseline and three weeks after the intervention. Comparing pre-treatment and values obtained at the third week, significant improvements were found in intensity of pain, neuropathic pain, functionality, and range of motion in both groups (*p* < 0.05). The diathermy plus exercises group had significantly better intensity of pain than the control group at the end of the three weeks (*p* < 0.01). The addition of diathermy by emission of radiofrequency to the therapeutic knee exercise protocol is more effective than a therapeutic exercise protocol alone in the relief of intensity of pain in patients with patellofemoral pain in the immediate post-treatment follow-up compared with baseline scores.

## 1. Introduction

Patellofemoral pain syndrome is a very common musculoskeletal dysfunction, characterized by pain in the anterior surface of the knee that often tends to chronicity [1]. Although it affects all population groups, a higher incidence has been observed in adolescents and young adults [2]. Pain while walking upstairs and downstairs, squatting, running, or sitting for a long time is referred to by most patients with patellofemoral pain syndrome [1,3]. Even though it has traditionally been associated with cartilage damage, knee osteoarthritis and elevated body mass index, researchers have shown no relation with these aspects [4,5]. According to some authors, the compression forces implicated in these activities could explain these symptoms [6]; however, some other authors’ views differ, pointing to unknown causes for patellofemoral pain [7]. A wide variety of pathologies can present similar signs and symptoms as patellofemoral pain syndrome, and for this reason, the term is used to describe any pain in the anterior surface of the knee [8]. Patellofemoral pain syndrome could precede patellofemoral osteoarthritis, a condition that may require surgical treatment and total knee replacement [9,10].

Non-surgery-based treatments are frequent, outshining physiotherapy as the most common approach [3,11]. The physiotherapy treatments include quadriceps strengthening to improve the active stability of the patella in the femoral trochlea, strengthening of the hip muscles, manual therapy, taping for patellar realignment, stretching, and therapeutic exercises [3,11,12,13,14,15,16]. Even though these treatments seem to produce benefits in patellofemoral pain syndrome, there is no evidence that one treatment modality is better than another intervention for any subgroup of patients [11,14]. Only therapeutic exercises have consistent evidence to support their recommendation [11,15,16]. Clinical guidelines also advise against the use of physical agents, including in this sense ultrasound, cryotherapy, sonophoresis, electrical stimulation, and laser [11]. However, no studies about the use of radiofrequency diathermy based on capacitive-dielectric energy transmission were considered due to the lack of them. 

Monopolar dielectric diathermy by emission of radiofrequency (MDR) is an endogenous thermotherapy, which consists in the emission of high frequency electromagnetic signals via an isolated electrode that transfers energy to soft tissues containing electrolytes: muscles, vascular, or lymphatic tissues [17]. This modality has a documented capacity to increase the local temperature of a tissue in order to stimulate its metabolism and reduce pain by a control gate mechanism [18,19]. Furthermore, some radiofrequency-based diathermy techniques have demonstrated to have more effects on intramuscular blood flow, tissue metabolism, pain and inflammation, muscle spasms, cell activity, and elasticity [17,18,19,20], than other physical agents, such as pulsed shortwave therapy [19].

Although other diathermy methods have documented its capacity to reduce pain in patients with numerous degenerative and inflammatory orthopedic problems, such as in low back pain or in shoulder impingement syndrome [21,22], evidence about the use of monopolar dielectric diathermy in knee pain is scarce [23,24,25]. Considering therapeutic knee and hip exercises as the most consistent approach for treating patellofemoral pain syndrome [3,11], and the hypothesis that this technique could promote pain and functional recovery, the purpose of this study was to compare the effectiveness of adding MDR to a therapeutic exercise protocol versus a therapeutic exercise protocol in the intensity of pain, probability of neuropathic pain, functionality, and range of movement of patients with patellofemoral pain syndrome.

## 2. Materials and Methods

### 2.1. Study Design

A prospective single-blind randomized controlled clinical trial was conducted, in which the researcher in charge of collecting the data from patients remained blind to the treatment applied to each participant. The trial was properly registered at ClinicalTrials.gov identifier NCT04538508. The investigation protocol was designed following the Helsinki Declaration and the Good Clinical Practice guidelines, according to CONSORT Standards and considering all the clinical regulations for research in humans. In this regard, all participants were appropriately informed about the study and their rights before signing the informed consent form accepting to participate. The research protocol was approved by the Ethics Committee of Virgen de la Macarena Hospital, Seville (CEI 1696-N-17).

### 2.2. Participants

A total of 120 participants were initially recruited for the study, who were between 18 and 65 years of age, diagnosed with patellofemoral pain syndrome, and who were not currently undergoing any type of treatment. To be eligible, patients had to meet the following inclusion criteria: (1) patients between 18 and 65 years of age; (2) referred pain in the anterior surface of the knee of 30 mm or over in the Visual Analogue Scale (VAS) during the previous three months; (3) without radiological findings compatible with osteoarthritis; (4) without sensitivity to patellar tendon or iliotibial band palpation; (5) scoring below 45 in the psychological apprehension scale (PPAS) [25]; (6) not having previously received radiofrequency diathermy treatment. The PPAS is a validated tool, reliable, and easy to use in evaluating the apprehension of the participants to receive electrical stimulation therapy [25]. The exclusion criteria included were (1) any contraindication for the use of diathermy by the emission of radiofrequency (tumours, use of implanted electronic devices as pacemakers, thrombophlebitis or deep venous thrombosis, pregnancy, active process of tuberculosis, fever, infected wounds, osteomyelitis, and rheumatoid arthritis) [17,26,27,28]; (2) having received a corticoid or hyaluronic acid injections treatment; (3) having reduced cognition or communication abilities; (4) or being currently involved in a medical-legal dispute. The use of basic analgesic drugs was allowed in order to avoid introducing significant changes in their treatment, but it was recorded to control possible changes that could influence in the final results. 

Sample size calculation was based on the detection of (1) an improvement of 15% in self-perceived pain intensity [29]; (2) a difference of >9 points in Lower Extremity Functionality Score at inter-group comparison after the treatment [30]; and >10 points in the Kujala Score. Considering a one-tail hypothesis, an alpha value of 0.05, a desired power of 95%, and a medium effect size (r^2^ = 0.25), and a 10% drop-out at follow-up, the desired sample size was calculated to be 30 participants per group (G* Power, version 3.1.9.2, Heinrich-Heine-Universität Düsseldorf, Düsseldorf, Germany). 

### 2.3. Outcomes Measures

**Figure 1:**
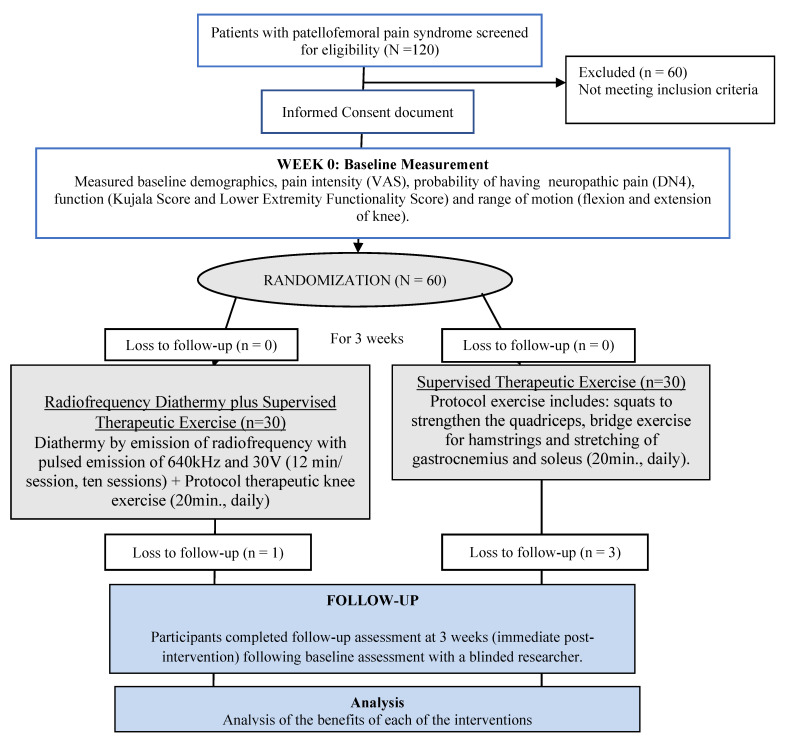
Design and flow of participants through the trial. Abbreviations: VAS = Visual Analogue Scale [32,33,34]; DN4 = Douleur Neuropathique-4 items [34,35].

All 60 participants provided demographic and clinical information, and also completed a number of self-report measures and underwent a physical examination, performed by an assessor blinded to the treatment allocation of the patients. Outcome measures were assessed before the first treatment session (baseline data), and immediately after treatment (at the third week) (Figure 1). Demographic measurements of weight, body mass index, metabolic age, and fat mass were performed with a Body Composition Analyser DC 430MA (III) device Japanese Technology (Tanita Europe B.V., Amsterdam, The Netherlands) [31]. 

The Visual Analogue Scale (VAS) was used to assess the patients’ current level of pain and the highest and lowest level of pain experienced in the preceding 24 h. A score of 00 would mean “no pain” and 10 “extreme and insufferable pain” [32]. The minimal clinically important differences (MCID) for VAS were determined as a variation of 15–20% [33] or a reduction of 2 in VAS after the intervention [34]. 

The Douleur Neuropathique-4 items (DN4) questionnaire, consisting of 10 items [34,35], was used to identify patients who had a high probability of having a neuropathic pain component. The scores of the individual items are added to obtain a maximum total score of 10, with a cut-off point ≥4.

The patient-reported measures of lower limb functional status were the Kujala Function Score and lower extremity functionality score. The Kujala Function Score is a 13-item self-administered questionnaire that regards symptomatology in people with patellofemoral pain syndrome, with a variable ordinal response format. The total scores range from 0 to 100 [36]. This tool presents a Cronbach’s alpha of 0.8 and test-retest Intraclass Correlation Coefficient (ICC) of 0.99 for its Spanish version [37]. Lastly, the lower extremity functionality score is a questionnaire which contains 20 questions to evaluate the function of the lower limb in patients who present local disorders [38]. The maximum possible score was 80 points, which indicated very high function, while 0 points indicated very low function. This questionnaire presents a high internal consistency (Cronbach’s alpha of 0.989) and an excellent test-retest reliability (ICC = 0.998, 95%) for the Spanish-speakers version [39].

The passive range of motion (ROM) in flexion and extension was measured with a conventional two-leg goniometer (angular measurement), which has shown high intratester reliability (ICC = 0.996, range 0.953–0.955 for both flexion and extension) and intertester (ICC range 0.959–0.970 for flexion and 0.85–0.898 for extension) [39].

### 2.4. Interventions

After the initial evaluation, 60 participants (considering a 1:1) with patellofemoral pain syndrome were randomly assigned to receive either MDR plus therapeutic knee exercise (experimental group) or therapeutic knee exercise alone (control group). Concealed allocation was performed using an external website (http://www.randomization.com; accessed on 6 September 2020) before the start of data collection by a researcher not involved in the recruitment or treatment of patients. One researcher distributed the randomized allocation of participants using opaque envelopes, with the participants being unaware of their selection. Another blinded researcher collected outcome measurements at baseline and immediately after the last treatment.

All participants received three weeks of the intervention. Diathermy treatment was performed across three weeks, consisting of ten treatment sessions in total (the first week comprised five sessions, the second week three sessions, and the third week two sessions), while therapeutic exercises for the knee were performed daily. Each session of therapeutic exercises lasted 20 min, and, in the case of the MDR group, another 12 min of diathermy were added before the exercise protocol. Both groups were treated by a physical therapist with more than 20 years of experience in the interventions. 

The details of the interventions are provided below.

#### 2.4.1. MDR plus Therapeutic Exercise

**Figure 2:**
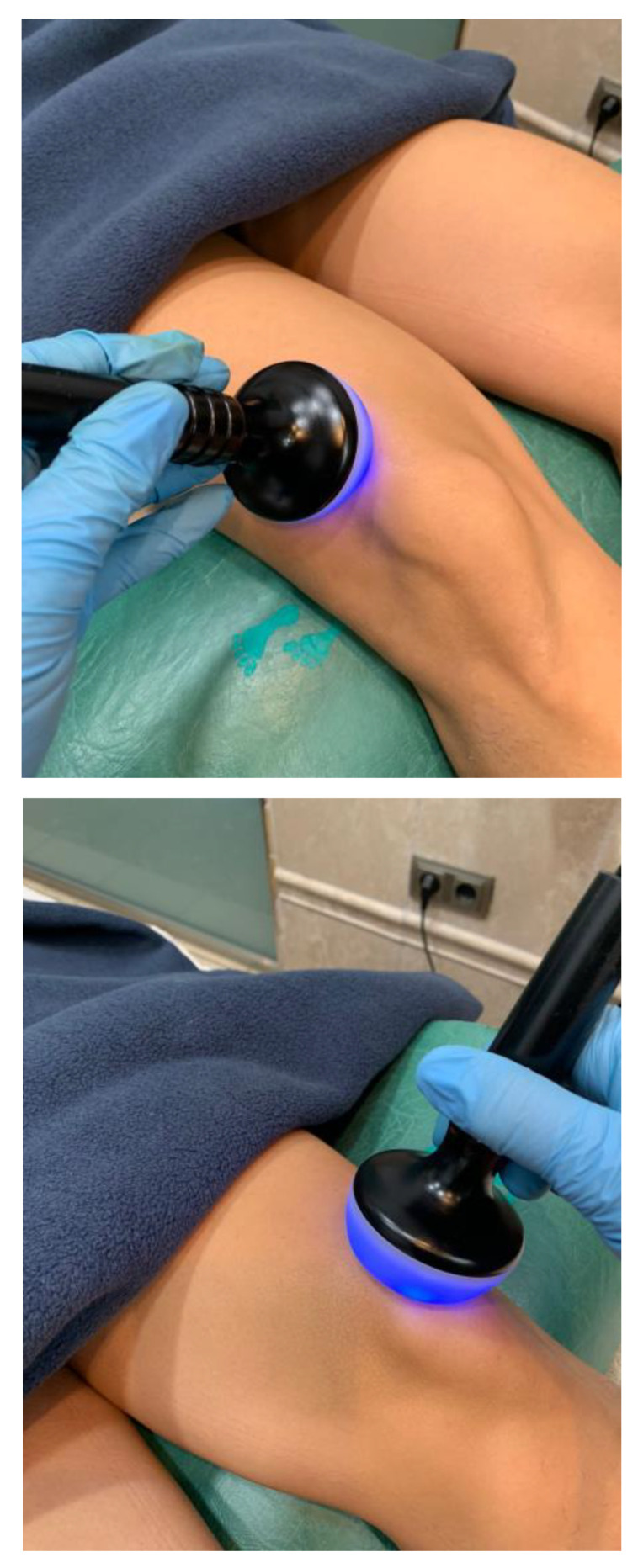
Monopolar dielectric diathermy by emission of radiofrequency.

In the MDR plus therapeutic exercise group, the participants received the same exercise protocol than the therapeutic exercise group, but prior to the exercise protocol. The participants received 12 min of monopolar dielectric diathermy by emission of radiofrequency with an ABD Modular^®^ device (Biotronic^®^, Granada, Spain), in pulsed emissions of 640 kHz and 30 V in dynamic application, with a continuous rotation and translational movement on the anterior surface of the knee (Figure 2). 

Five milliliters of almond oil were used to improve gliding along the twelve-minute application of MDR [17,25]. The use of almond oil as transfer substance was due to the fact that a dielectric transmission device was used instead of a resistive-capacitive one; this allows the energy to be focused on depth, minimizing heating tissues on the surface [17].

#### 2.4.2. Therapeutic Exercise

All participants were instructed to perform the exercise protocol for knee stability, according to the recommendations of Van Der Heijden [16]. Participants performed the therapeutic exercises over three weeks, under the supervision of a physiotherapist. The protocol included the following:

1. Squats for concentric strengthening of quadriceps: standing with the affected knee in the maximum degree of flexion that the subject was able to achieve, the knee was slowly straightened to its full extension. Series: 3. Repetitions: 20.
2. Squats for eccentric strengthening of quadriceps: standing with the affected knee, which was slowly flexed to the maximum degree of flexion that the subject was able to achieve. Series: 3. Repetitions: 20.
3. Side step: consisted in lowering the good leg over the side edge of the step without touching the ground. Series: 3. Repetitions: 20
4. Bridge exercise for hamstrings: the patient lied supine on a mat, with the knees bent, the soles of the feet well supported, and the heels at a distance of half a foot from the gluteus. From this position, the patient had to raise the pelvis towards the ceiling. Series: 3. Repetitions: 20 s.
5. Clam exercise for gluteus medius: the patient lied on their side with the knees bent and keeping the feet together. Then, the patient had to raise the knee of the top leg, opening the legs up so that the legs made the shape of a clam. Series: 3. Repetitions: 20 s.
6. Soleus Stretch Standing: standing with the affected leg back, both knees bent, keeping the heels on the floor, turned slightly out, leaning the body towards the wall until the stretch was felt in the lower calf. Series: 3. Repetitions: 1 min.
7. Gastrocnemius Stretch Standing: same as the previous stretch but with the affected knee extended. Series: 3. Repetitions: 1 min.

The exercises were performed during approximately 20 min according to the patients’ possibilities, not exceeding 3 of pain in VAS and including one minute of rest among each series. Those patients who reported a pain sensation of 3 or greater reduced the number of repetitions and increased the rest time, thus reducing the level of resistance requested. The progression was performed according to the patient’s sensations.

### 2.5. Statistical Analysis

An assessor blinded to the treatment allocation conducted the statistical analysis using SPSS statistical software, version 27.0. Data were reported as mean (standard deviation) and confidence intervals (IC 95%). Firstly, the normal distribution of variables was verified by the Kolgomorov–Smirnov test, after a descriptive analysis. Levene test was used to assess the homogeneity of variances. Linearity was assessed by bivariate dispersion graphics of residual values observed from the expected values. 

Baseline demographic and clinical variables were examined between both groups, with independent Student’s t-test for continuous data and χ^2^ tests of independence for categorical data. Separate 2 × 2 mixed model ANOVA with time (baseline and post- treatment) as the within-subjects factor, and group (MDR plus therapeutic exercise or therapeutic exercise) was used to determine the effects of the treatment. Effect size was tested using Cohen’s d. An effect size <0.2 reflects a negligible difference, between ≥0.2 and ≤0.5 a small difference, between ≥0.5 and ≤0.8 a moderate difference, and ≥0.8 a large difference. Eta squared (η^2^) was also used to calculate the effect size (small, 0.01 ≤ η^2^ < 0.06; medium, 0.06 ≤ η^2^ < 0.14; and large, η^2^ > 0.14). A *p*-value less than 0.05 was considered to indicate a statistically significant difference. 

## 3. Results

Four general practitioners (blinded to group allocations and assessment) from a primary care health center of the Andalusian Health Service (Seville, Spain) recruited 120 participants between August and September 2020. After referral by a general practitioner, patients were interviewed face to face by another blinded researcher to check that they met the inclusion and exclusion criteria. A total of 60 participants with patellofemoral pain syndrome met the inclusion criteria and were recruited for the clinical trial (Figure 1). After the inclusion phase, four subjects withdrew from the study because they missed almost one session of treatment and 56 participants were thus included in the study, 27 men and 29 women [mean age: 43.18 (5.7) years]. They were randomly assigned either to MDR plus therapeutic exercise group (n = 29) or to therapeutic exercise group (n = 27). Out of the knees treated, 29 were right (52%) while the remaining 27 were left (48%). The mean (SD) for demographic characteristics and differences between groups at baseline are shown in Table 1 (*p* > 0.05 for all). 

Within group analysis showed a significant improvement from baseline values for all subscales in the MDR plus therapeutic exercise group (change score: VAS = 4.8, DN4 = 4.1, Kujala Score = 19.2, Lower Extremity Functionality Score = 22.4, flexion = 15.7°; *p* < 0.001); only the range of movement in extension obtained a *p* = 0.031 (change score extension = 1.0). Although the therapeutic exercise group also experienced changes in all subscales, except for extension (*p* = 0.161), these differences were smaller than in the MDR plus exercise group (change score: VAS = 0.9, DN4 = 1.8, Kujala Score = 19.7, Lower Extremity Functionality Score = 14.2, flexion = 8.8°; *p* < 0.002). Table 2 includes baseline and post-treatment outcomes, as well as the between-groups mean differences and effect size. 

An ANOVA test showed statistically significant differences between groups with respect to intensity of pain (F_1,54_ = 37.79, *p* = 0.000, η^2^ = 0.41), and probability of neuropathic pain (F_1,54_ = 4.23, *p* = 0.045, η^2^ = 0.07). Although no significant differences between groups were found for disability and range of movement, the results showed greater improvement in Lower Extremity Functionality Score in the MDR plus therapeutic exercise group (change score: 22.4) than in the therapeutic exercise group (change score: 14.2) at post-treatment follow-up. No significant differences between groups were found for Kujala Score (F_1,54_ = 1.4, *p* = 0.242, η^2^ = 0.025), Lower Extremity Functionality Score (F_1,54_ = 0.18, *p* = 0.67, η^2^ = 0.003), and range of movement (Flexion: F_1,54_ = 3.44, *p* = 0.069, η^2^ = 0.06; Extension F_1, 54_ = 0.01, *p* = 0.91, η^2^ = 0.000).

## 4. Discussion

The main finding of the present study is that the addition of MDR to therapeutic exercise produces a greater improvement in intensity of pain and probability of neuropathic pain than only supervised exercises in patients with patellofemoral pain in the immediate post-treatment follow-up compared with baseline scores. Moreover, the addition of MDR reduces disability to a greater degree than only exercise at short term. These findings are clinically very relevant as exercises have shown good results for improving function, but moderate for short-term pain reduction [12]. Through the data of this study, useful evidence to support the use of diathermy by radiofrequency in addition to therapeutic exercises for the knee has been obtained.

Benefits of exercises have been observed related to functionality in patellofemoral pain syndrome [14,16]. These improvements related to movement had been explained by the effects of exercise on central nervous system neuroplasticity, which enhances the subject’s capacity to respond to new demands with functional adaptations [40]. For this reason, despite the lack of consensus about exercise in patellofemoral pain syndrome, the existing evidence is consistent enough to be the most recommended approach.

Considering this, our hypothesis was based on the point that MDR plus therapeutic exercise for the knee could make a difference on pain and recovery time reduction. According to the results of this study, pain decreased in 48 in VAS (*p* < 0.001) when diathermy by radiofrequency was added to therapeutic exercises. This result agrees with those referred by previous studies such as the one of Kumaran and Watson [23] in knee osteoarthritis, who obtained 40 pain improvements in VAS, and the one of Albornoz et al. [24] in patellofemoral pain syndrome, where the diathermy group obtained a difference of 53 with respect to the control group. Summarizing, it must be outstood that the addition of MDR to therapeutic exercises obtained greater reductions in pain than therapeutic exercises alone. However, it must be considered that most of the studies about exercise for patellofemoral pain syndrome lasted for months [11], so the three-week intervention used in this study according to the local public health system could have reduced the potential benefits of exercise in this condition. In addition, and given that the patients in the diathermy group could not be blinded, a positive expectation of success in pain treatment or a placebo effect could have been created. This could be the reason for the differences between the groups. However, some authors have determined that each medical treatment takes place in the context of individual expectations and previous experiences [41,42]. Among the inclusion criteria, we determined that the participants had to have never received radiofrequency diathermy in order to minimize bias; although, it would be advisable to investigate its possible effects in future studies.

Regarding function, no significant differences were observed between the MDR plus therapeutic exercise group versus the therapeutic exercise alone group, both obtaining significant improvements at post-treatment follow-up. However, adding MDR to the exercises seems to produce greater improvements in function than performing therapeutic exercises alone (differences between groups: Kujala Score 4.4; Lower Extremity Functionality Score: 5.3). We believe that these differences can be explained by the relation between pain intensity and kinesiophobia [43]. Previous studies have demonstrated that the presence of fear of movement may influence treatment outcome. Studies show that in people with chronic musculoskeletal pain, fear of physical exercise or movement is due to the common assumption of increased pain or injury, and this has been associated with increased pain intensity and disability [44,45,46,47]. There is no doubt that physical exercise has been an important component in the treatment of pain in both groups [48]; although, the diathermy group could have had better results in terms of pain and thus better function. However, this study has not evaluated the pain–kinesiophobia–function relationship in patients with patellofemoral pain syndrome. Another explanation for not having observed this more clearly could be that participants do not have enough time in the three weeks of intervention to assess subjective improvements in their daily function, as most of the studies use a larger period of intervention of four, six, or eight weeks [16,20]. Due to the fact that range of motion is not as subjective as Kujala or Lower Extremity Functionality Score, it is understandable that more clear improvements were assessed in this outcome. 

Regarding range of motion in flexion, statistically significant improvements were observed in patients of the diathermy group compared with those in the therapeutic exercise group. This could be explained in different ways; on the one hand, it is known that patients suffering from greater pain usually show higher levels of fear to movement, so the pain relief may have improved the range of motion [49]. While, on the other hand, the results of the study are consistent with those of Szabo et al. [50], who stated that the recovery protocol of combining therapeutic physical exercises with endogenous thermotherapy processes has beneficial results in recovering flexion and reducing pain. Furthermore, Ribeiro et al. [51] demonstrated that diathermy therapy is a good complementary method in the treatment of musculoskeletal disorders, which should be incorporated into the rehabilitation program or used in isolation, with both short- and long-term effects.

For all the above, it could be recommended to always use exercise as the first approach for patellofemoral pain syndrome since its effects are more than demonstrated in all the outcome measures. Adding MDR could also be useful, effective, and safe to obtain higher pain reductions and reduce the time of recovery, as it has been shown in various musculoskeletal medical disorders such as sports-type injuries, in low back pain, and in urology [21,52,53,54]. Due to its thermotherapy implications, the diathermy therapy encourages the therapeutic procedures of wounded tissues without unwanted elevation of skin temperature [20]. However, more studies combining both therapies in patellofemoral pain syndrome are needed to confirm that a reduction in the healing process occurs.

The main weakness of this study is related to the lack of a placebo group of MDR. The difficulty to solve this lies in the impossibility of producing a thermal sensation similar to diathermy without heating or promoting metabolism of the tissue. Although the use of non-emission devices is accepted, participants’ distrust could easily increase with these devices due to the lack of sensation and therefore they may suspect the sham. Another limitation is the lack of a long-term follow-up, which was not viable owing to the different places of residence of most of participants and the pandemic situation. 

Future research should evaluate the long-term results and implement longer treatment programs. In addition, physiological responses such as vascularity, deep muscle temperature, motor unit recruitment, etc., should be assessed. On the other hand, it should also be assessed whether the results are satisfactory enough for the application of treatment to be profitable at the clinical level.

In terms of clinical relevance, this randomized clinical trial has shown significant short-term improvements in pain intensity, probability of neuropathic pain, and range of motion in flexion in patients with patellofemoral pain with a three-week MDR adjunct to exercise therapeutics. The study highlights the potential benefits of radiofrequency emission monopolar dielectric diathermy in the treatment of patellofemoral pain syndrome and provides baseline data for future research in other musculoskeletal disorders.

## 5. Conclusions

The addition of MDR to therapeutic knee exercises is more effective than only therapeutic exercises at reducing intensity of pain, probability of having neuropathic pain, and range of motion in flexion in patients with patellofemoral pain syndrome. The main finding of the present study is that the addition of MDR to therapeutic exercise produces a greater improvement in intensity of pain and probability of neuropathic pain than only supervised exercises in patients with patellofemoral pain in the immediate post-treatment follow-up compared with baseline scores.

## Figures and Tables

**Table 1:** Baseline characteristics of participants in the study groups.

Variables	Total Sample (n = 56)	Diathermy Plus Therapeutic Exercise Group (n = 29)	Therapeutic Exercise Group (n = 27)	*p* Value *
Mean age (years)	43.2 (5.72)	42.3 (15.52)	51 (10.89)	0.083
Height (cm)	168.6 (11.48)	167 (11.39)	169 (11.70)	0.533
Weight (kg)	79.3 (16.03)	76.1 (13.61)	82.3 (17.82)	0.118
Sex				
Female	29	11	18	
Male	27	16	11	
Body Mass Index	27.8 (4.18)	27.1 (3.98)	28.6 (4.32)	0.183
Fat Mass (%)	30.1 (9.41)	29.4 (10.61)	30.8 (8.06)	0.578
Metabolic age (years)	50 (19.37)	45 (19.91)	55 (17.79)	0.062
PPAS	23.64 (7.98)	23.51 (4.15)	23.97 (5.17)	0.143
Educational Level				0.184
School level	19	9	10	
Bachelor level	20	8	12	
University level	17	12	5	

* Values are expressed as absolute frequency for categorical variables and as mean (SD) for continuous variables (N = 56). *p* associated with student’s *t*-test for independent samples in continuous variables and chi-square in categorical variables. Abbreviation: PPAS = Personal Psychological Apprehension Scale.

**Table 2:** Baseline, immediate post-treatment follow-up, and change score between groups for intensity of pain, neuropathic pain, functionality, and range of movement of the knee.

Outcomes	Baseline (Week 0)	Post-Intervention (Week 3)	Mean Differences and CI between Groups	Cohen’s d
Patient-reported outcomes				
VAS (0–10)				
Diathermy plus therapeutic exercise	5.7 (1.91)	0.9 (1.46)	4.0 (3.2, 4.7) **	1.45
Therapeutic exercise	5.8 (1.21)	4.9 (3.31)		
DN4 (0–10)				
Diathermy plus therapeutic exercise	4.3 (2.07)	0.2 (0.51)	1.7 (1.2,2.2) **	1.86
Therapeutic exercise	3.8 (1.28)	1.9 (1.16)		
KUJALA (0–100)				
Diathermy plus therapeutic exercise	54.4 (19.53)	73.7 (15.83)	−4.4 (−12.3, 3.3)	0.25
Therapeutic exercise	49.5 (14.95)	69.2 (13.17)		
LEFS (0–80)				
Diathermy plus therapeutic exercise	42.3 (8.75)	64.8 (13.02)	−5.3 (−11.3, 0.7)	0.39
Therapeutic exercise	45.3 (15.21)	59.5 (9.04)		
Passive ROM (º)				
Flexion				
Diathermy plus therapeutic exercise	118.3 (10.62)	133.9 (7.36)	−8.0 (−13.4, −2.6) *	0.70
Therapeutic exercise	117 (11.02)	125.9 (12.32)		
Extension				
Diathermy plus therapeutic exercise	1.2 (2.55)	0.2 (0.92)	0.7 (−0.3, 1.8)	0.32
Therapeutic exercise	0.5 (1.60)	0.9 (2.78)		

* Values are expressed as means ± standard deviation for baseline and three weeks post-treatment and as mean score change (95% confidence interval) for between-group values. * Indicates statistically significant between-groups differences (*p* < 0.05). ** Indicates statistically significant between-groups differences (*p* < 0.001). Abbreviations: DN4 = Douleur Neuropathique-4 items; LEFS = Lower Extremity Functionality Score; ROM = Range of Movement; VAS = Visual Analogue Scale.

## Data Availability

The data presented in this study are available on request from the corresponding authors.
